# Errors in Byline and Author Contributions

**DOI:** 10.1001/jamanetworkopen.2026.43594

**Published:** 2026-10-15

In the Original Investigation titled “Self-Pay Prices and Financial Assistance Programs for Urology Appointments”^1^ published August 25, 2026, there was an error in Arnav Surpur’s name in the byline and in the Author Contributions. This article has been corrected.^1^
